# HDAC1 disrupts the tricarboxylic acid (TCA) cycle through the deacetylation of Nur77 and promotes inflammation in ischemia-reperfusion mice

**DOI:** 10.1038/s41420-023-01308-1

**Published:** 2023-01-18

**Authors:** Zhenhua Wu, Yunpeng Bai, Yujuan Qi, Chao Chang, Yan Jiao, Yaobang Bai, Zhigang Guo

**Affiliations:** 1 Academy of Medical Engineering and Translational Medicine, Tianjin University, 300222 Tianjin, China; 2 ICU, Department of Cardiac Surgery, Tianjin Chest Hospital, 300222 Tianjin, China; 3 Department of Cardiac Surgery, Tianjin Chest Hospital, 300222 Tianjin, China

**Keywords:** Molecular biology, Biochemistry

## Abstract

Histone deacetylase enzymes (HDACs) regulate protein acetylation. HDAC1 is known to enhance ischemia/reperfusion (I/R) injury, but its underlying mechanism(s) of action have not been defined. Here, in vivo mouse models of myocardial I/R were used to investigate the role of HDAC1 during I/R myocardial injury. We show that HDAC1 enhances the inflammatory responses of I/R mice. Using a constructed macrophage H/R (hypoxia/ regeneration) injury model (Raw264.7 cells), we identified Nur77 as a HDAC1 target in macrophages. Nur77 deficient macrophages failed to downregulate IDH1 (isocitrate dehydrogenase 1) and accumulated succinic acid and other tricarboxylic acid (TCA) cycle-derived metabolites in a glutamine-independent manner. These data show that the inhibition of HDAC1 ameliorates H/R-inflammation in macrophages through the regulation of Nur77 and the TCA cycle.

## Introduction

Histone deacetylases (HDACs) play a key role in a range of pathophysiological conditions [1]. HDACs comprise classes I, II, III, and IV [2] and remove acetyl groups from lysine residues in histones and other cellular proteins to regulate gene expression [3]. The functional relevance of HDAC1 in cardiovascular disease remains uncharacterized.

Ischemic heart disease (IHD), specifically acute myocardial infarction, is a leading cause of mortality [4, 5]. Despite the acute nature of ischemia and re-perfusion injury (I/R), harmful effects of IHD on the myocardium are observed [6]. Low energy and oxygen levels lead to the depletion of ATP, the disruption of ionic homeostasis, increased glycolysis, disturbed oxidative phosphorylation in mitochondria, increased mitochondrial membrane permeability (MMP), and decreased myocardial contractility [7–9]. Previous studies have shown that the inhibition of HDAC1 preceding I/R injury is protective to left ventricular activity and the survival of myocardial cells [10–12].

Nur77 (NR4A1) is a transcription factor first observed in pheochromocytoma (PC12) tumor cells [13]. Nur77 participates in cell maturity, proliferation, and differentiation through its cooperation with epidermal growth factor (EGF) and nerve growth factor (NGF) [14]. Tumor cell apoptosis is facilitated by the relocalisation of Nur77 to the mitochondrial membrane [15, 16]. At the post-transcriptional level, Nur77 is regulated by acetylation. Histone deacetylase 1 (HDAC1) deactivates Nur77 leading to its ubiquitination [17]. HDAC1 mediated Nor1 and Nur77 inhibition promotes the survival of myeloid leukemia blast cells and stem cells [18]. The role of Nur77 and HDAC1 during I/R injury is poorly defined.

The activation of macrophages through inflammatory stimulation leads to mitochondrial metabolic re-programming and the production of proinflammatory cytokines. Metabolic reprogramming is marked by the down-regulation of IDH1, which regulates a range of inflammatory processes including macrophage polarization [19]. In this study, in vivo mouse models of myocardial I/R and a hypoxia/ regeneration (H/R) injury model in Raw264.7 cells were designed to investigate the role of HDAC1 during myocardial injury.

## Results

### HDAC1 promotes inflammation post-I/R

**Fig. 1:**
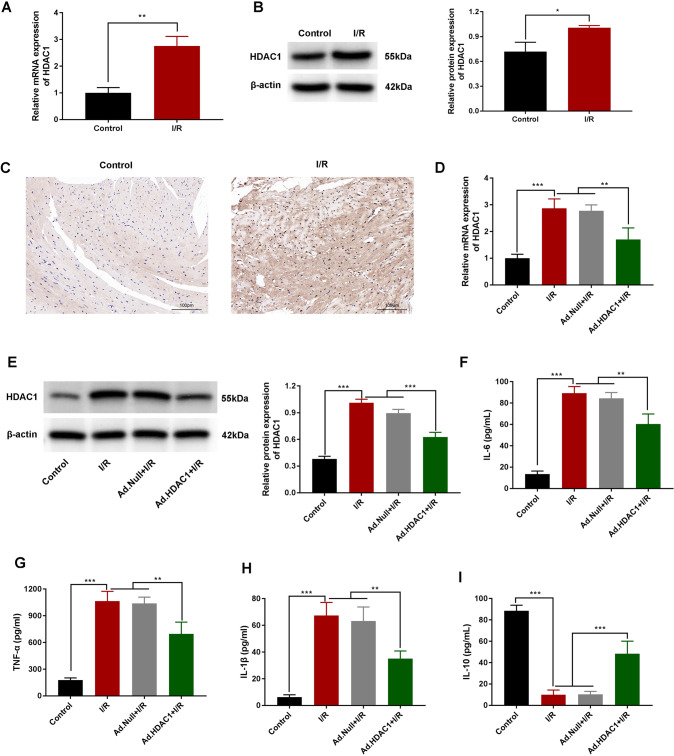
HDAC1 promotes inflammation post-I/R. **A** qRT-PCR analysis of HDAC1 mRNA in control and I/R mice. **B** HDAC1 expression determined via western blotting. **C** HDAC1 expression in the infarct area analyzed by IHC. **D** qRT-PCR and **E** western blot analysis of HDAC1 expression in I/R mice injected with HDAC1-silencing adenovirus HDAC1 (Ad.HDAC1). **F**–**I** IL-6, TNF-a, IL-1β, and IL-10 levels in the supernatants of I/R mice assessed via ELISA. Data are the mean ± SD, *n* = 3; **P < 0.01. ***P < 0.001.

To investigate the role of HDAC1 in I/R, we constructed a myocardial ischemia-reperfusion mouse model. QRT-PCR and western blot analysis showed increased expression of HDAC1 compared to the control group (*P* < 0.01, *P* < 0.001 (Fig. 1A, B). Immunohistochemical analysis revealed higher levels of HDAC1 expression in infarct areas (Fig. 1C).

To further analyze the function(s) of HDAC1 in I/R models, mice were intramuscularly injected with empty-vector (Ad.Null) or an adenovirus silencing HDAC1 (Ad.HDAC1). As shown in Fig. 1D--E, HDAC1 expression was comparable between the Ad. Null+I/R and I/R groups, but decreased in the Ad group (*P* < 0.01, *P* < 0.001). When collected plasma samples were assayed for cytokine release by EILSA, higher levels of proinflammatory TNF-a, IL-6, and IL-1β were observed in the I/R group compared to the control (*P* < 0.001) or Ad groups. The Null+I/R group showed no difference to the I/R group. IL-6, TNF-a, and IL-1β concentrations were lower in the Ad-HDAC1 + I/R group compared to the I/R group (*P* < 0.01, *P* < 0.001) (Fig. 1F–H). In contrast, the levels of IL-10 in I/R mice were significantly lower than the control group, but increased in the Ad-HDAC1 + I/R group (*P* < 0.01, *P* < 0.001; Fig. 1I). These results confirmed that HDAC1 promotes inflammation in I/R mouse models.

### Inhibition of HDAC1 improves H/R-induced macrophage inflammatory responses

**Fig. 2:**
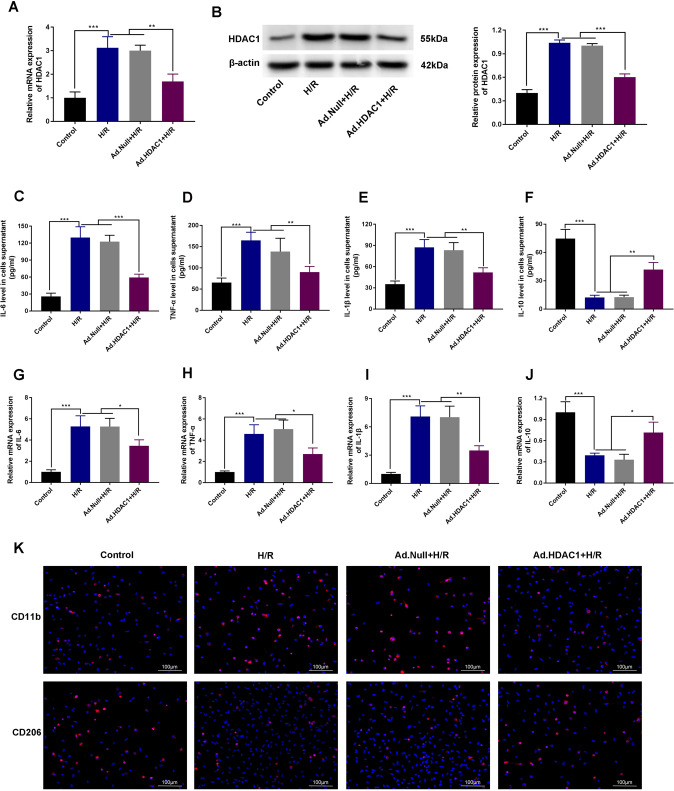
Inhibition of HDAC1 ameliorates H/R induced inflammation in macrophages. **A** qRT-PCR and **B** western blot analysis of HDAC1 expression in H/R Raw264.7 cells transfected with Ad.HDAC1. **C**–**F** ELISA analysis of cytokine levels. **G**–**J** IL-6, IL-10, TNF-α, and IL-1β gene expression determined by qRT-PCR. **K** Immunofluorescence analysis of CD206 and CD11b expression. *n* = 3. Data are the mean ± SD. *P < 0.05, **P < 0.01, ***P < 0.001.

We constructed an in vitro H/R injury model in RAW264.3 cells (hypoxia, 8 h; reoxygenation, 16 h). As shown in Fig. 2A, B, RAW264.3 cells in the H/R group showed higher HDAC1 expression compared to the control group (*P* < 0.001). HDAC1 levels were significantly lower in H/R mice treated with Ad.HDAC1 (*P* < 0.01, *P* < 0.001). ELISA analysis revealed lower levels of IL-6, IL-1β and TNF-α and increased levels of IL-10 in both H/R and Ad-HDAC1 groups compared to Null+H/R and control groups (*P* < 0.001; Fig. 2C–F). The levels of IL-6, TNF-α, and IL-1β were significantly lower in the H/R group treated with Ad. HDAC1, whilst the levels of IL-10 increased (*P* < 0.01, *P* < 0.001). All data were confirmed at the mRNA level via qRT-PCR analysis (*P* < 0.05, *P* < 0.01, *P* < 0.001, Fig. 2G–J). Immunofluorescent analysis showed increased expression of CD11b (a marker of M1 macrophages) and lower levels of CD206 (a marker of M2 macrophages) in H/R and Ad.Null+H/R groups compared to the control group. The Ad.HDAC1 + H/R group showed lower levels of CD11b and increased CD206 expression (Fig. 2K). Collectively, these data suggest that the inhibition of HDAC1 ameliorates macrophage inflammation in response to H/R.

### HDAC1 regulates metabolic reprogramming in macrophages following H/R

**Fig. 3:**
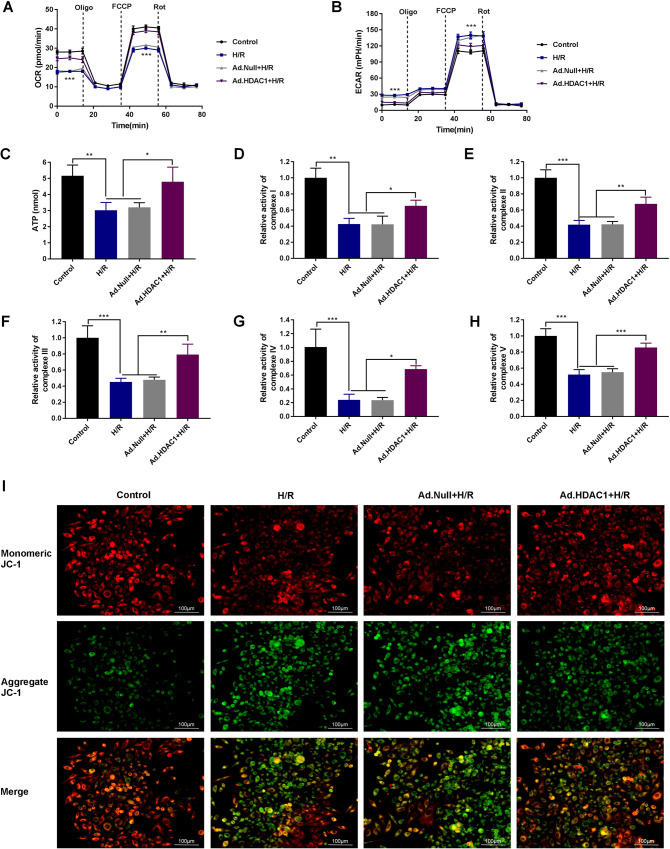
HDAC1 regulates the metabolic reprogramming of macrophages following H/R. **A** Seahorse assays showing decreased oxidative consumption rates (OCR) in H/R (blue) compared to controls (black). Increased OCR in Ad.HDAC1 + H/R (purple) compared to H/R (blue) in response to oligomycin. **B** Seahorse assays demonstrating increased extracellular acidification rates (ECAR) in H/R (blue) vs. control groups (black), and decreased ECAR in Ad.HDAC1 + I/R (purple) vs. H/R groups (blue) in response to oligomycin. **C** ATP production in H/R following Ad.HDAC1 transfection. Complexes I (**D**), II (**E**), III (**F**), IV (**G**), and V (**H**) activity in RAW264.3 cells. **I** MMP in RAW264.3 cells. High-intensity red fluorescence and green fluorescence denote increased and decreased membrane potentials, respectively (×400, bar = 100 μm). *n* = 3. All data are mean ± SD. *P < 0.05, **P < 0.01, ***P < 0.001.

Metabolic reprogramming in macrophages has been linked to polarization and inflammation. Seahorse metabolic assays were performed to establish if the altered energy production was due to the changes in glycolysis or oxidative phosphorylation. RAW264.3 cells in the H/R group showed a significantly lower oxidative consumption rate (OCR) compared to control RAW264.3 cells. Ad.HDAC1 transfection led to a higher OCR compared to Ad. Null+H/R, indicative of increased levels of oxidative phosphorylation (Fig. 3A). In H/R cells, the extracellular acidification rate (ECAR) was higher compared to the control group. Ad.HDAC1 caused a decrease in the ECAR compared to the Ad.HDAC1 + H/R group, indicating reduced glycolytic capacity (Fig. 3B). ATP assays in RAW264.3 cells in the H/R group showed significantly lower levels of ATP production compared to the control group (*P* < 0.001). Ad.HDAC1 led to increased ATP production compared to H/R and Ad. Null+H/R groups (*P* < 0.01, Fig. 3C). In the H/R group, the activity of the respiratory chain complex I-V decreased in RAW264.3 cells, but increased in Ad.HDAC1 cells (*P* < 0.05, *P* < 0.01, *P* < 0.001; Fig. 3D–H). Assessment of the MMP using JC-1 (Fig. 3I) revealed a lower MMP in H/R RAW264.3 cells that was reversed by Ad.HDAC1. Together, these data suggest that the inhibition of HDAC1 leads to lower levels of mitochondrial damage.

### HDAC1 participates in mitochondrial metabolic reprogramming through the regulation of Nur77 deacetylation

**Fig. 4:**
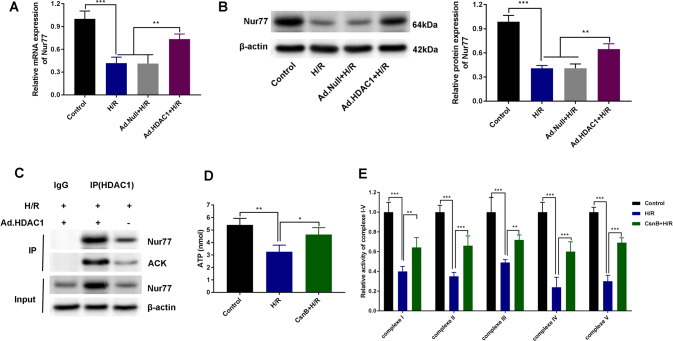
HDAC1 participates in mitochondrial metabolic reprogramming through the regulation of Nur77 deacetylation. **A** qRT-PCR and **B** western blot analysis of Nur77 expression in H/R Raw264.7 cells transfected with Ad.HDAC1. **C** Acetylation (AC-K) of Nur77 regulated by HDAC1 evaluated through Co-IP assays. **D** ATP production in H/R following CsnB transfection. **E** Complex I, II, III, IV, and V activity in RAW264.3 cells (*n* = 3). Data represent the mean ± SD. *P < 0.05, **P < 0.01, ***P < 0.001.

As shown in Fig. 4AB, H/R and Ad. Null+H/R groups showed weaker Nur77 mRNA and protein expression compared to the control group. The levels of Nur77 were significantly higher in H/R cells transfected with Ad.HDAC1 (*P* < 0.01, *P* < 0.001). To confirm whether the acetylation of Nur77 was regulated by HDAC1 following H/R intervention, HDAC1 Co-immunoprecipitation (CO-IP) assays were performed. We found that the levels of Nur77 acetylation were significantly higher in the AD.HDAC1 + H/R group (Fig. 4C). The activity of Nur77 was enhanced by CsnB which increased ATP production in the I/R group (*P* < 0.05; Fig. 4D). In addition, CsnB could restore the levels of complexes I, II, III, IV, and V in H/R RAW264.3 cells (*P* < 0.01, *P* < 0.001) (Fig. 4E).

### Nur77 participates in inflammatory responses through enhanced transcriptional activity of IDH1

**Fig. 5:**
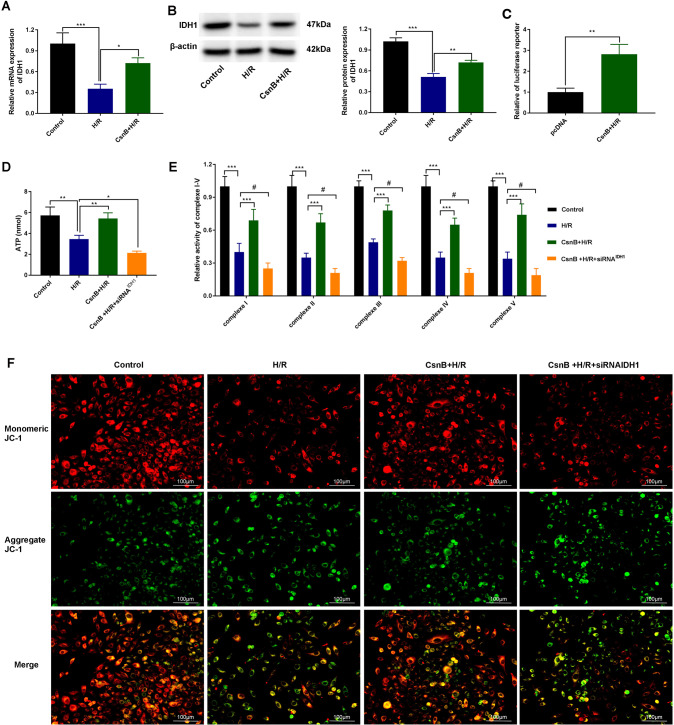
Nur77 promotes inflammation through the regulation of IDH1 in H/R cells. **A** qRT-PCR and **B** western blot analysis of IDH1 expression in H/R Raw264.7 cells transfected with CsnB. **C** Luciferase reporter assays in CsnB transfected H/R Raw264.7 cells. **D** ATP production in H/R RAW264.3 cells co-transfected with CsnB and siRNA^IDH1^. **E** Complex I, II, III, IV, and V in H/R RAW264.3 cells following co-transfection of CsnB and siRNA^IDH1^. **F** Mitochondrial membrane potential assayed via JC-1 staining in H/R RAW264.3 cells co-transfected with CsnB and siRNA^IDH1^ (×400, bar = 100 μm). *n* = 3. Data represent the mean ± SD. *P < 0.05, **P < 0.01, ***P < 0.001, ^#^*P* < 0.05.

We next sought to determine whether Nur77 regulates the transcriptional activity of IDH1 in H/R RAW264.3 cells. Western blot and qRT-PCR analysis showed lower expression of IDH in the H/R group. However, IDH expression was significantly higher in the H/R group + CsnB (*P* < 0.05, *P* < 0.001; Fig. 5A, B). Dual-luciferase reporter assays showed increased luciferase activity in the CsnB+H/R group compared to the empty vector group (*P* < 0.001, Fig. 5C). Furthermore, IHD1 siRNA (siRNA^IDH1^) when co-transfected with CsnB into H/R RAW264.3 cells showed lower levels of ATP production and reduced complex I–V activity compared to the control group (*P* < 0.01, *P* < 0.001). CsnB alone enhanced ATP production and complex I-V activity in the H/R group (*P* < 0.001). siRNA^IDH1^ further restored the CsnB induced changes in H/R RAW264.3 cells (*P* < 0.05, Fig. 5D, E). JC-1 staining following IHC analysis showed similar effects of siRNA^IDH1^ on the MMP (Fig. 5F). Together, these findings demonstrate that Nur77 participates in inflammatory responses through its effects on the transcriptional activity of IDH1 in H/R cells.

### Nur77 activator (CsnB) improves inflammatory responses following I/R in vivo

**Fig. 6:**
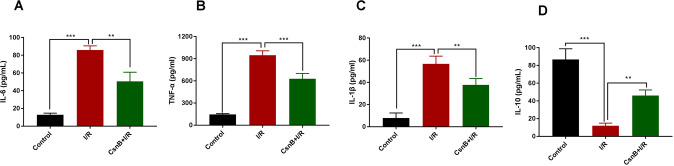
CsnB alleviates inflammatory responses post-I/R in vivo. **A**–**D** Pro-inflammatory and anti-inflammatory cytokine secretions were identified via ELISA. *n* = 3. Data are the mean ± SD. **P < 0.01, ***P < 0.001.

To further investigate the role of Nur77 in the regulation of I/R by HDAC1, I/R mice were treated with CsnB. ELISA analysis in the I/R group showed increased concentrations of IL-6, TNF-α, and IL-1β and a decreased concentration of IL-10 in the plasma of mice (*P* < 0.001). As shown in Fig. 6A–D, CsnB treatment could reverse these changes (*P* < 0.01, *P* < 0.001), revealing its ability to alleviate inflammatory responses post-I/R.

## Discussion

I/R injury occurs following coronary artery disease for which the treatment options remain limited [9, 20]. HDAC inhibitors can preserve myocardial function in a range of animal models [21]. Here, we developed an I/R mouse and H/R injury model in RAW264.3 cells to demonstrate that I/R-induced myocardial injury can be partially alleviated by HDAC1 inhibition.

Previous studies have reported the detrimental effects of HDACs on I/R injury [10, 12, 21]. We found that HDAC1 promotes inflammation in I/R mice and that HDAC1 inhibition suppresses inflammation. We further demonstrated that H/R leads to inflammation and mitochondrial damage.

Macrophages are key to innate immune responses due to their role in tissue development, host defenses and cellular homeostasis [22]. In activated macrophages, the inflammatory response leads to profound reprogramming during cellular metabolism [23]. M1 and M2 macrophages display distinct functions [24]. M1 macrophages express high levels of CD11b [25, 26] and produce proinflammatory cytokines. M2 macrophages are characterized by high CD206 and IL-10 expression [27, 28]. We found that HDAC1 inhibition increased the polarization of M2 macrophages but reduced M1 polarization in H/R RAW264.3 cells. Furthermore, the inhibition of HDAC1 increased both the glycolytic capacity and energy efficiency of RAW264.3 cells, increasing ATP production and mitochondrial complex I-V activity. The inhibition of HDAC1 also decreased the H/R-induced loss of MMP. Oxidative phosphorylation and metabolic reprogramming are characteristic of activated inflammatory M1 macrophages [29]. Glycolysis, oxidative phosphorylation, pentose phosphate pathway, fatty acid oxidation and the metabolism of amino acids represent key metabolic pathways [30, 31]. Ischemic preconditioning activates mitochondrial Src, regulating complex I activity and the levels of mitochondrial reactive oxygen species (ROS) to counter myocardial I/R [32]. In line with these studies, mitochondrial dysfunction and oxidative stress were observed in H/R RAW264.3 cells, which increased following HDAC1 silencing. HDAC1 therefore contributed to oxidative stress and mitochondrial dysfunction through the progression of I/R.

HDAC1 prevents the acetylation and subsequent expression of Nur77 [18]. In RAW264.3 cells, HDAC1 was found to interact with Nur77 and inhibit its acetylation. The inhibition of HDAC1 enhanced the levels of AcK, a promotor driven by Nur77, implying that the inhibition of HDAC1 facilitated the hyperacetylation of Nur77. CsnB has been identified as a ligand for Nur77 [33, 34] and promoted oxidative stress in H/R RAW264.3 cells. Moreover, CsnB could improve the inflammatory response in I/R mice. Collectively, these data suggest that HDAC1 participates in mitochondrial metabolic reprogramming through the regulation of Nur77 deacetylation.

IDH1 participates in the TCA cycle, catalyzing oxidative isocitrate decarboxylation for the production of alpha-ketoglutarate (AKG) and CO_2_ [35]. Activated macrophages are glycolytic and produce high levels of ROS and succinate [36]. We found that siRNA^IDH1^ enhanced the effects of CsnB in H/R RAW264.3 cells (*P* < 0.05, Fig. 5D, E). These data suggest that Nur77 participates in inflammatory responses through its regulation of the transcriptional activity of IDH1.

In summary, we identify the HDAC-mediated deacetylation of Nur77 as a novel mechanism that is critical for macrophage function following I/R injury. This identifies the HDAC-Nur77 axis as potential therapeutic target for much-needed I/R therapeutics.

## Materials and methods

### Animals studies and I/R grouping

Six-week-old BALB/c nude mice (22–25 g weight) were purchased from Charles River, Beijing, China. Mice were fed an identical diet and provided free access to water. All procedures were approved by the Animal Ethics Committee of the Tianjin Chest Hospital (TJCH-2022002). Mice were divided into 4 groups (*n* = 6 per-group): [1] Control; [2] I/R; [3] Ad.HDAC1 + I/R; and [4] Ad.Null+I/R groups. Mice were euthanatized using 40 mg/kg sodium pentobarbital (i.p.). Blood and tissue samples were collected for subsequent analysis.

### In vivo regional I/R surgery

Anesthesia, intubation and ventilation were performed as previously described [37, 38]. Regional I/R was performed through the ligation of LAD for 30 min and its release for a single day with a left lateral thoracotomy.

### Cell culture and H/R

Raw264.7 cells were purchased from ATCC and maintained in DMEM (Gibco, USA) supplemented with 10% FBS and 100 U/mL streptomycin-penicillin. Raw264.7 cells were assigned to the following groups: [1] Control; [2] H/R; [3] Ad.Null+H/R; [4] Ad.HDAC1 + H/R and [5] CsnB+H/R (Nur77 activator, Cytosporine B (CsnB) 10 μg/ml). For H/R, cells were cultured in hypoxic buffer (5% CO2; 1% O2; 94% N2) for 30 min at 37 °C in a hypoxia/anaerobic incubator [39]. Cells were then incubated in DMEM + 10% FBS for 1 h to reoxygenate the cells.

### Adenovirus transduction

Mice were infected with 2.4 × 10^7^ PFU/ml recombinant adenovirus for HDAC1 silencing (Genchem, Changzhou, China). For I/R, the heart was exposed between the 4th and 5th ribs of the left chest, and microinjections were performed into the left ventricle anterior wall using a 30 G (Gauge) needle (Ad.Null or Ad.HDAC1). For in vitro experiments, Raw264.7 cells were seeded into 6-well plates and transfected with lentiviral plasmids carrying Ad.HDAC1 using Lipofectamine 3000 (Invitrogen, USA).

### Macrophage polarization assays

Immunofluorescence analyses were performed as previously described [40, 41]. Briefly, Raw264.7 cells were seeded into 24 well plates and fixed for 30 min in 4% paraformaldehyde. Cells were permeabilsed in 0.05% Triton X-100 for 10 min, blocked in 1% BSA in PBS for 10 min and probed with anti-CD11b (1:100) and anti-CD206 (1:200) primary antibodies overnight at 4 °C. Cells were labeled with fluorescently conjugated goat anti-mouse IgG (Abcam, UK). Nuclei were stained with DAPI. Cells were imaged on a confocal laser scanning microscopy (CLSM, LSM 510 META; Germany).

### RT-PCR

Cells were lysed in TRIzol (Invitrogen, Waltham, MA, USA) and total RNA was isolated. Samples were DNase-I treated and subject to first-strand cDNA synthesis using oligo-dT primers (Invitrogen, USA) and M-MuLV reverse transcriptase (Fermentas, USA). Primers for RT-PCR were as follows: HDAC1 (F: 5′-CCGCATGACTCATAATTTGCTG-3′, R: 5′-ATTGGCTTTGTGAGGGCGATA-3′), Nur77 (F: 5′- GTTGATGTTCCCGCCTTTGCC-3′, R: 5′- TCAGAAAGACAATGTGTCCAT-3′), IDH1 (F: 5′-TGCCACCAACGACCAAGTCA-3′, R: 5′-TGTGTTGAGATGGACGCCTATTTG-3′), and β-actin (reference gene) (F: 5′-CATGTACGTTGCTATCCAGGC-3′, R: 5′-CTCCTTAATGTCACGCACGAT-3′).

### Western blotting

Cells were washed in PBS and lysed in Radio-Immunoprecipitation Assay (RIPA) buffer on ice. Lysates were clarified by centrifugation and proteins were resolved by SDS-PAGE electrophoresis. Samples were transferred onto nitrocellulose membranes and probed with the following primary antibodies: anti-HDAC1, anti-Nur77, and anti-IDH1 (1:1000) for 24 h at 4 °C. Membranes were labeled with horseradish peroxidase (HRP)-conjugated anti-mouse antibodies for 1 h. Luminal agent was used for protein detection (SC-2048, Santa Cruz Biotechnology, CA, USA).

### ELISA

Blood samples were collected and serum IL-6, TNF-a, IL-10, and IL-1β levels were evaluated by ELISA (USCN Life Science, Inc., China). Absorbances were read on a microplate reader (Bio-Tek Instruments, Inc., USA).

### Metabolic parameters

Cells were treated with Seahorse XF assay medium (pH = 7.4) supplemented with 2 mM glutamine, 1 mM pyruvate, and 10 mM glucose. Cells were incubated in CO_2_ free conditions for 1 h hour at 37 °C. Oxygen consumption (OCR) and extracellular acidification (ECAR) rates were measured following the addition of glucose, 2-deoxy-d-glucose (2DG) and oligomycin. Cells were further assessed following treatment with carbonyl cyanide 4-trifluoromethoxyphenylhydrazone (FCCP), oligomycin, glucose, 2-DG, rotenone, and antimycin A (Rot/AA). Adenosine triphosphate (ATP) levels and metabolic parameters were calculated using a Seahorse XF96 Analyzer (Agilent, Santa Clara, CA, USA).

### Mitochondrial respiratory chain complex activity

Mitochondrial respiratory activity was measured using commercial kits (ab110419, Abcam, Cambridge, UK) as per the manufacturer’s instructions. Absorbances were measured on a BioRad microplate reader (BioRad, Hercules, CA).

### Mitochondrial membrane potential

JC-1 was used as a marker of MMP. When Dwm is high, JC-1 produces red fluorescence. Green fluorescence is produced by JC-1 monomers. For actin staining, cells were permeabilized in 0.1% Triton X-100 for 5 min, blocked in 10% goat serum in PBS for 1 h and labeled with mouse anti-a-actin (Sigma, St. Louis, USA, 1:1000) and Cy3 labeled secondary antibodies for 24 h at 4 °C. Nuclei were counterstained with DAPI.

### Co-immunoprecipitation analysis

Cells were washed in PBS and resuspended in BC300 buffer (0.2 mM EDTA, 0.2 mM PMSF, 20 mM Tris-HCl, 300 mM NaCl, 0.2% Tween-20, and 10% glycerol), followed by sonication for 10 min on ice. Lysates were incubated with primary antibodies (anti-Nur77, ab283264, Abcam; acetylated-lysine antibody (ACK) #9441, CST) for 24 h at 4 °C. Anti-Rabbit IgG antibodies were added as a negative control. Lysates were incubated with A/G agarose beads (Cell Signaling Technology, USA) for 3 h and centrifuged. Beads were washed in BC300 buffer, boiled and analyzed on 15% SDS-PAGE gels.

### Statistical analysis

SPSS 20.2 and GraphPad Prism 7 were used for data analysis. Data represent the mean ± SD. A Student’s *t*-test (two-tailed) or a one-way ANOVA were used for single or multiple group comparisons, respectively. *P* < 0.05 was deemed statistically significant.

## Supplementary information

original data files

## Data Availability

All the data used to support the findings of this study are included within the article.
