# Supplementary figures and images for: HDAC1 disrupts the tricarboxylic acid (TCA) cycle through the deacetylation of Nur77 and promotes inflammation in ischemia-reperfusion mice
Source: Cell Death Discov. 2023 Jan 18;9:10. doi: 10.1038/s41420-023-01308-1 (PMC9849262; doi:10.1038/s41420-023-01308-1)

Figure 1B

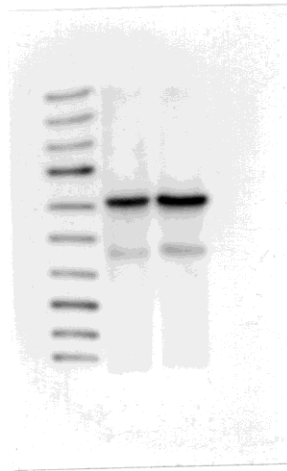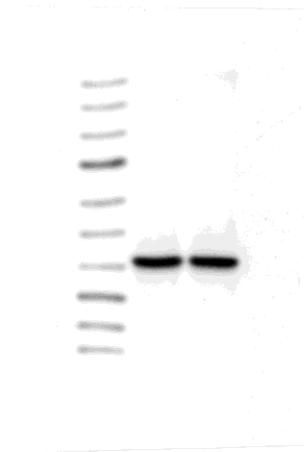

Figure 1E

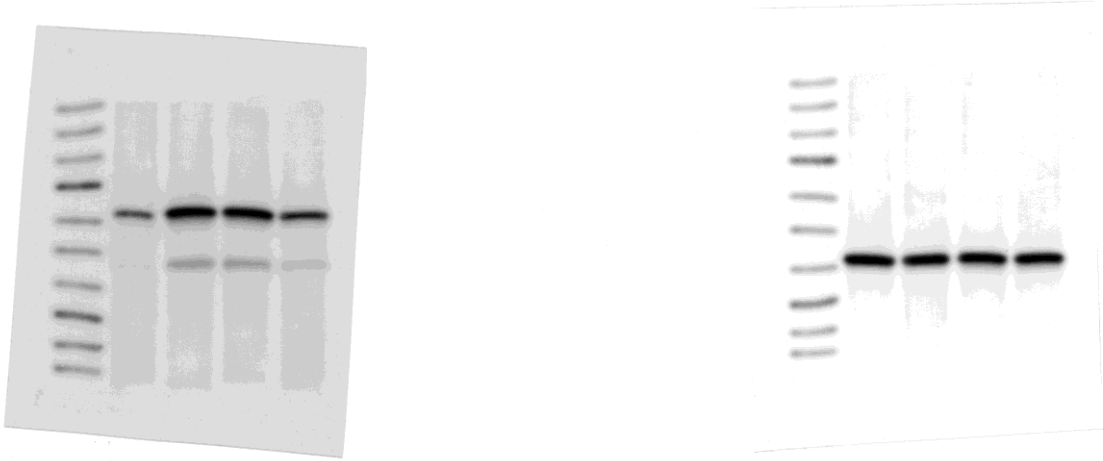

Figure 2B

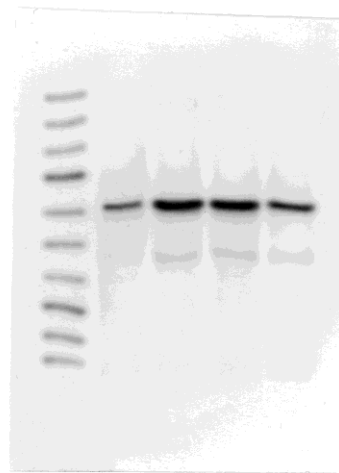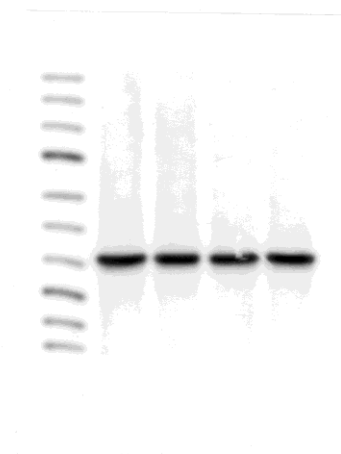

Figure 4B

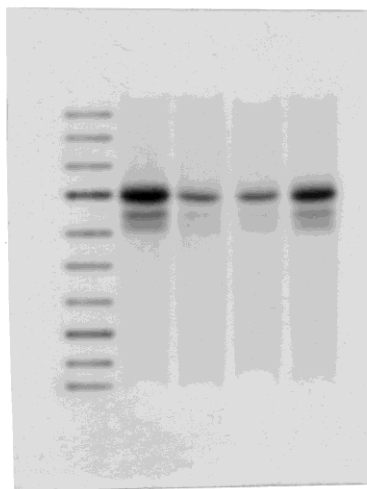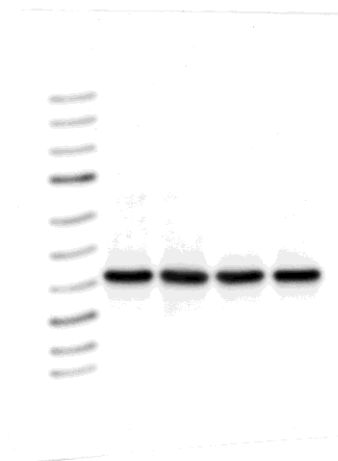

Figure 4C

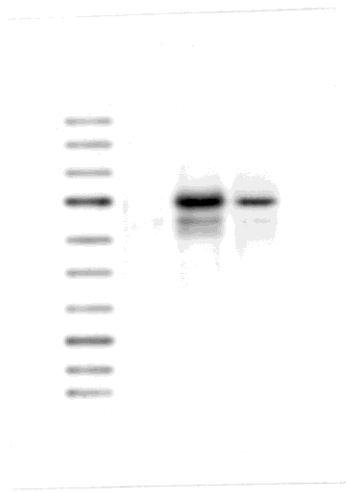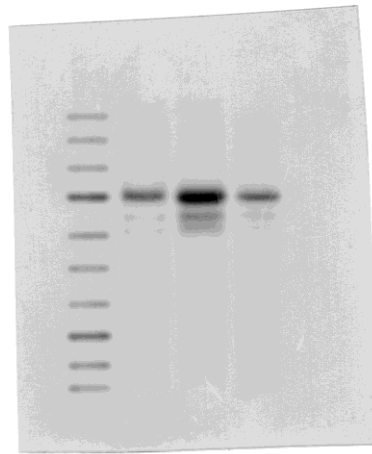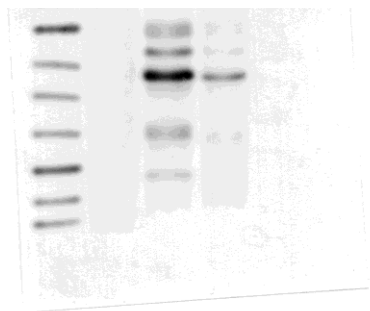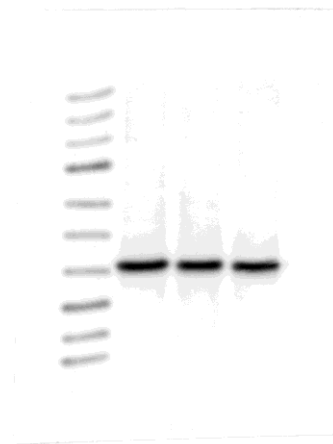

Figure 5B

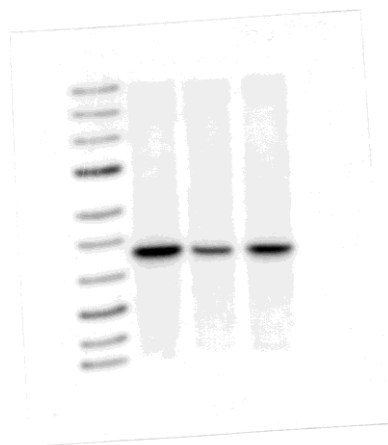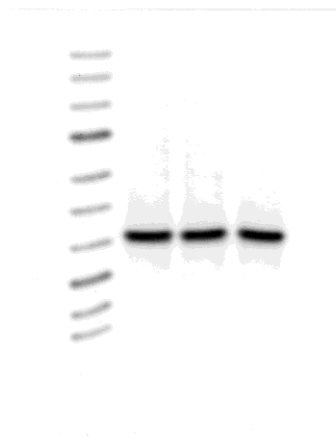

Supplement: Supplementary file 1 — original data files [file 41420_2023_1308_MOESM1_ESM.pdf]
